# UNMET NEEDS OF OLDER FEMALE VETERANS AND GENDER DIFFERENCES USING THE PROSPECTIVE HERO CARE SURVEY DATA

**DOI:** 10.1093/geroni/igad104.3682

**Published:** 2023-12-21

**Authors:** Stuti Dang, Pranjal Tyagi, Sandra Garcia, Erin Bouldin, Ranak Trivedi, Orna Intrator, Mary Jo Pugh, Luci Leykum

**Affiliations:** University of Miami, Miami, Florida, United States; South Florida VA Foundation for Research and Education (SFVAFRE), Miami, Florida, United States; University of Miami, Miami, Florida, United States; Decision-Enhancement and Analytic Sciences Center (IDEAS 2.0.), Salt Lake City, Utah, United States; Stanford University, Stanford, California, United States; Canandaigua VAMC and University of Rochester, Canandaigua, New York, United States; VA Salt Lake City Health Care System, Salt Lake City, Utah, United States; South Texas Veterans Health Care System, San Antonio, Texas, United States

## Abstract

Aging female Veterans face unique healthcare needs which are yet to be well-described. We analyzed round one data from HERO CARE, a prospective survey, to describe the medical, psychological, and social unmet needs of female Veterans and compare them with those of male Veterans. We received survey responses from 8,056 community-dwelling Veterans (227 (2.8%) females and 7,829 (97.2%) males) from San Antonio, Palo Alto, Miami, Salt Lake City Veterans Affairs (VA) Medical Centers, and Veteran Integrated Service Network 8 in July 2021. We compared difference in needs between the female and male respondents using chi-square tests and difference in age in years using t-tests. Respondents were classified as having an unmet need if they responded that they needed “a little” or “a lot more help” for specific needs. Female respondents were 74.3 (SD: 14.5) years-old on average, and 7.9% Hispanic, 15.4% non-Hispanic Black, 72.7% non-Hispanic White. Male respondents were older 80.3 (SD: 9.8) years-old on average (p< 0.01), with no significant difference in race/ethnicity. Female Veterans reported unmet needs in ADLs (14.1%), IADLs (40.1%), nursing tasks (7.1%), pain management (17.6%), communicating with the healthcare team (10.6%), and social needs like legal or housing (16.3%). All unmet needs were like those of male Veterans (all p>0.05). Among female Veterans, 20.1% screened positive for anxiety (GAD-2 score >2) and 22.8% for depression (PHQ-2 score >2), compared to male Veterans: 15.2% (p< 0.01) and 21.6% (p=0.41), respectively. Routine assessment of aging female Veterans, specifically focusing on mental health and IADLs, may better address their needs.

